# Expulsion of the Tape-worm by the Oil of the Male Fern

**Published:** 1851-03

**Authors:** 


*Expulsion of the Tape-worm by the Oil of the Male Fern.*—Dr. Budd, of King's College Hospital, London, has been lately making some interesting experiments with an old anthelmintic, the oil of the male fern, in expelling the tape-worm, and reports a very gratifying success. Mr. Pollard, M. R. C. S., of Halifax, has also used the oil of the filix mas, with marked success.—*Abridged from London Lancet, 7th and 21st Dec.,* 1850.

